# Inhibition of melanogenesis by jineol from *Scolopendra subspinipes mutilans* via MAP-Kinase mediated MITF downregulation and the proteasomal degradation of tyrosinase

**DOI:** 10.1038/srep45858

**Published:** 2017-04-10

**Authors:** Md Badrul Alam, Vivek K. Bajpai, JungIn Lee, Peijun Zhao, Jung-Hee Byeon, Jeong-Sic Ra, Rajib Majumder, Jong Sung Lee, Jung-In Yoon, Irfan A. Rather, Yong-Ha Park, Kangmin Kim, MinKyun Na, Sang-Han Lee

**Affiliations:** 1 Department of Food Science and Biotechnology, Graduate School, Kyungpook National University, Daegu 41566, Korea; 2 Department of Applied Microbiology and Biotechnology, Microbiome Laboratory, Yeungnam University, Gyeongsan, Gyeongbuk 38541, Korea; 3 College of Pharmacy, Chungnam National University, Daejeon 34134, Korea; 4 Department of Biological Sciences, Macquarie University, Sydney, NSW 2109, Australia; 5 Bio-security and Food Safety, NSW Department of Primary Industries, Elizabeth Macarthur Agricultural Institute (EMAI), Menangle, NSW 2567, Australia; 6 Kcellbio, Seoulsoop Kolon Digital Tower, Seongsuil-ro-4-gil, Seongdong-gu 04713, Seoul, Korea; 7 Division of Biotechnology, College of Environmental and Bioresource Sciences, Chonbuk National University, 79 Gobong-ro, Iksan-si 570-752, Jeonbuk, Republic of Korea

## Abstract

In this study, the authors investigated the anti-melanogenic effects of 3,8-dihydroxyquinoline (jineol) isolated from *Scolopendra subspinipes mutilans*, the mechanisms responsible for its inhibition of melanogenesis in melan-a cells, and its antioxidant efficacy. Mushroom tyrosinase activities and melanin contents were determined in melan-a cells, and the protein and mRNA levels of MITF, tyrosinase, TYRP-1, and TYRP-2 were assessed. Jineol exhibited significant, concentration-dependent antioxidant effects as determined by DPPH, ABTS, CUPRAC, and FRAP assays. Jineol significantly inhibited mushroom tyrosinase activity by functioning as an uncompetitive inhibitor, and markedly inhibited melanin production and intracellular tyrosinase activity in melan-a cells. In addition, jineol abolished the expressions of tyrosinase, TYRP-1, TYRP-2, and MITF, thereby blocking melanin production and interfering with the phosphorylations of ERK1/2 and p38. Furthermore, specific inhibitors of ERK1/2 and p38 prevented melanogenesis inhibition by jineol, and the proteasome inhibitor (MG-132) prevented jineol-induced reductions in cellular tyrosinase levels. Taken together, jineol was found to stimulate MAP-kinase (ERK1/2 and p38) phosphorylation and the proteolytic degradation pathway, which led to the degradations of MITF and tyrosinase, and to suppress the productions of melanin.

Melanin, the major skin color determining factor, provides defense against the harmful effects of ultraviolet (UV)-induced skin damage^1^. Despite its advantages, abnormal melanin production is responsible for several skin diseases, such as, albinism, leukoplakia, melasma, freckles, moles, and lentigo^1^, and thus, the modulation of melanogenesis is viewed as an important strategy for treating abnormal skin pigmentation. Melanogenesis involves a series of enzymes, such as, tyrosinase, TYRP-1, and TYRP-2, and their transcription factors, microphthalamia-associated transcription factor (MITF), and diverse signal transduction pathways^2^. Tyrosinase (a copper containing enzyme) plays a key role in the synthesis of melanin, as it catalyzes the two rate-limiting reactions of melanogenesis, namely the hydroxylation of tyrosine to 3,4-dihydroxyphenylalanine (DOPA) (monophenolase) and the oxidation of DOPA to dopaquinone (diphenolase)^3^. In the absence of a thiol, such as, cysteine or glutathione, tyrosinase-related protein 2 (TYRP-2), acts as a dopachrome tautomerase, and rearranges dopachrome to 5,6-dihydroxyindole (DHI) or indole 5,6-quinone 2-carboxylic acid (DHICA). Furthermore, DHICA is oxidized by TYRP-1 into eumelanins during melanogenesis, and MITF, the prime transcription factor, can transcriptionally regulate the expressions of tyrosinase, TYRP-1, and TYRP-2^4^.

Ultra-violet radiation (UV) generates reactive oxygen species (ROS), including hydrogen peroxide (H_2_O_2_) and the hydroxyl (HO^•^), and superoxide (O_2_^•−^) -radicals in melanocytes and induces melanin biosynthesis, which primarily results in the production of pheomelanin^1^,^5^. Evidence indicates that the UV-induced generation of ROS and reactive nitrogen species (RNS) correlates with increased melanogenesis, possibly via the upregulation of tyrosinase at the protein and mRNA levels in melanocyte cells^6^. In addition, nitric oxide (NO) has been reported to up-regulate melanin production in melanocytes by activating the α-melanocyte stimulating hormone/melanocortin 1 receptor (α-MSH/MC1R) or MITF signaling pathway^7^,^8^. These observations suggest that the antioxidant-defense system might play a beneficial role by mitigating the detrimental effect of excessive melanin production induced by oxidant formation.

Melanogenesis is modulated by a variety of signal transduction pathways, such as, the cyclic adenosine monophosphate (cAMP)/protein kinase A pathway, which is viewed as the most important regulator of melanogenesis^9^. Furthermore, mitogen-activated protein kinase (MAPK) signaling via extracellular-signal-regulated kinases (ERK), c-Jun N-terminal kinase (JNK), and p38 MAPK, leads to MITF ubiquitination and degradation and plays a pivotal role in suppressing melanin production^2^.

Researchers continue to develop biologic reductants and tyrosinase inhibitors, such as, kojic acid, sulfite^10^, and arbutin^11^, to treat hyper-pigmentary disorders and ameriorate complexion discolorations. However, whitening products based on tyrosinase inhibitors have severe side effects, which include cellular toxicity, and poor stability in the presence of oxygen and water. Because they are considered safe and largely free from side effects, natural materials are now viewed as being suitable for the development of effective and safe skin depigmenting agents in the cosmetic research and development field.

Recently, natural products from insects have attracted attention in the cosmetic industry. CopA3 peptide derived from *Copris tripartitus* (the dung beetle) was utilized as a skin-friendly cosmetic^12^, and a novel antimicrobial peptide, scolopendrasin I, discovered in the centipede *Scolopendra subspinipes mutilans* (Scolopendridae) was developed as an atopic dermatitis cosmetic^13^. This centipede is a recognized medicinal resource for the traditional treatment of spasms, seizure, stroke-related hemiplegia, tetanus, and pain in China and Korea^14^,^15^. Our chemical investigations on *S. subspinipes mutilans* revealed the presence of quinoline alkaloids, including 3,8-dihydroxyquinoline (jineol), 2,8-dihydroxy-3,4-dimethoxyquinoline, and scolopendrine^16^. Jineol (3,8-dihydroxyquinoline) was first isolated from *S. subspinipes mutilans* in 1996^17^.

We hypothesized that jineol functionally associates with MITF to downregulate the expressions of the melanocyte-specific enzymes, tyrosinase, TYRP-1, and TYRP-2. In the present study, investigated whether jineol suppresses melanogenesis by inhibiting melanogenesis specific enzymes via its upstream effects and by acting as an antioxidant in melan-a cells. In addition, we investigated the involvement of the p38 and the ERK phosphorylation in the mechanism underlying melanogenesis inhibition by jineol in melan-a cells, also the jineol-induced proteasomal degradation of tyrosinase to confirm its inhibitory potential.

## Results

### Identification and characterization of jineol

**Figure 1:**
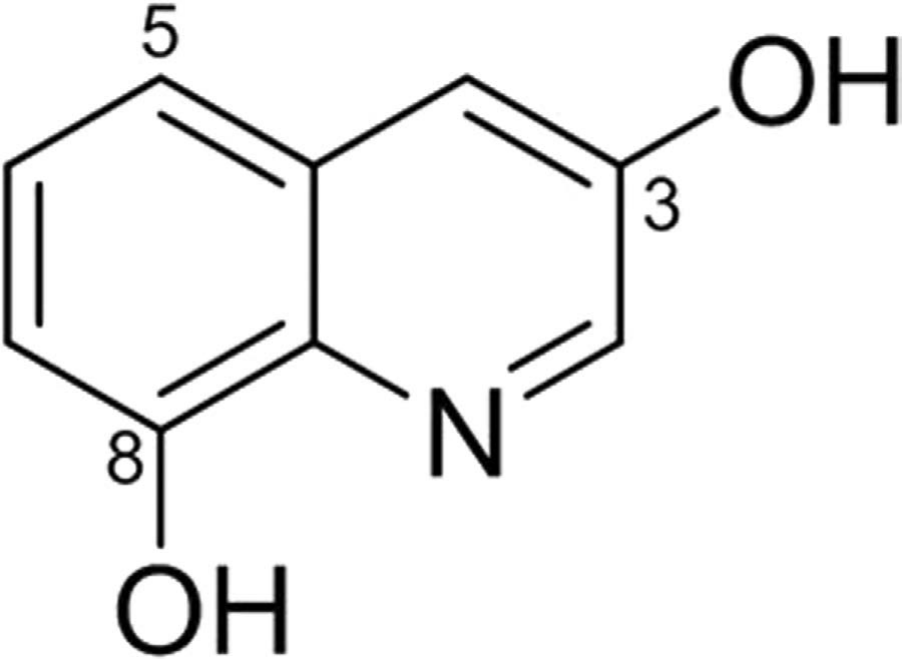
Chemical structure of jineol isolated from *Scolopendra subspinipes mutilans*.

^1^H NMR data showed ABX spin system at *δ*_H_ 7.29 (1 H, t, *J* = 8.0 Hz, H-6), 7.14 (1 H, d, *J* = 8.0 Hz, H-5), and 6.87 (1 H, d, *J* = 8.0 Hz, H-7) and a *meta*-coupled aromatic protons at *δ*_H_ 8.46 (1 H, d, *J* = 2.4 Hz, H-2), 7.42 (1 H, d, *J* = 2.4 Hz, H-4). Typical proton signals for 3-hydroxy quinoline alkaloid were observed at *δ*_H_ 8.46 (1 H, d, *J* = 2.4 Hz, H-2) and 7.42 (1 H, d, *J* = 2.4 Hz, H-4). Inspection of the ^13^C NMR spectrum revealed nine aromatic carbons: two oxygenated benzene signals at *δ*_C_ 153.0 (C-3), 154.2 (C-8), two quaternary carbons at *δ*_C_ 134.8 (C-8a), 131.8 (C-4a), and five aromatic signals at *δ*_C_ 142.3 (C-2), 129.0 (C-6), 117.9 (C-5), 117.2 (C-4), and 109.0 (C-7). Based on its NMR spectroscopic data analyses, the compound was identified as 3,8-dihydroxyquinoline (jineol)^17^ (Fig. 1).

### *In vitro* antioxidant activity of jineol

**Figure 2:**
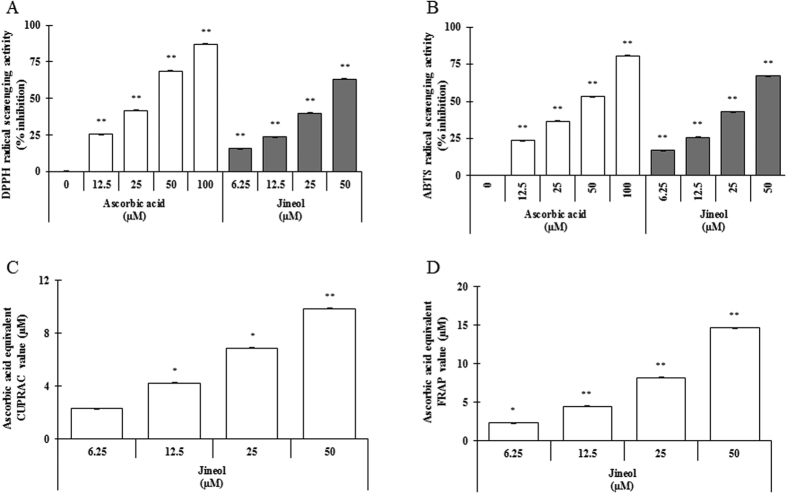
Antioxidant properties of jineol as determined by various *in vitro* antioxidant assays. DPPH radical scavenging activity (**A**), ABTS radical scavenging activity (**B**), CUPRAC activity (**C**), and FRAP activity (**D**) were analyzed as described in Materials and Methods. Each determination was made in triplicate, and results are represented as means ± SDs. *P < 0.05, **P < 0.01, versus non-treated controls by the Student’s t-test.

Antioxidants protect cells from oxidative stress, and antioxidant capacity may be defined as the ability to scavenge free radicals and reactive oxygen and nitrogen species by hydrogen or electron donation. To determine whether jineol has radical scavenging activities, we examined its ability to scavenge DPPH- and ABTS-radicals. Jineol significantly scavenged DPPH^·^ (a stable organic nitrogen radical) and ABTS^•+^, in a mixed electron and hydrogen atom transfer assay, in a dose-dependent manner (Fig. 2A and B). To confirm the electron-donating ability of jineol, we assessed its cupric-reducing antioxidant capacity (CUPRAC) and ferric-reducing antioxidant power (FRAP). Jineol was found to have strong reducing capacity and to act in a concentration-dependent manner (Fig. 2C and D), indicating it potently scavenges various free radicals by hydrogen atom transfer and electron donation. Pearson’s correlation analysis was performed to confirm its antioxidant and anti-melanogenic activities. Interestingly, the results obtained showed that antioxidant capacities of jineol ranked remarkable scores by exhibiting Pearson’s score as ρ = 0.989 for anti-tyrosinase activity, and ρ = 0.961 for anti-melanogenic activity (data not shown).

### Determination of anti-melanogenic effect using mushroom tyrosinase and kinetic parameters of the effects of jineol on the mono- and diphenolase activities of tyrosinase

**Figure 3:**
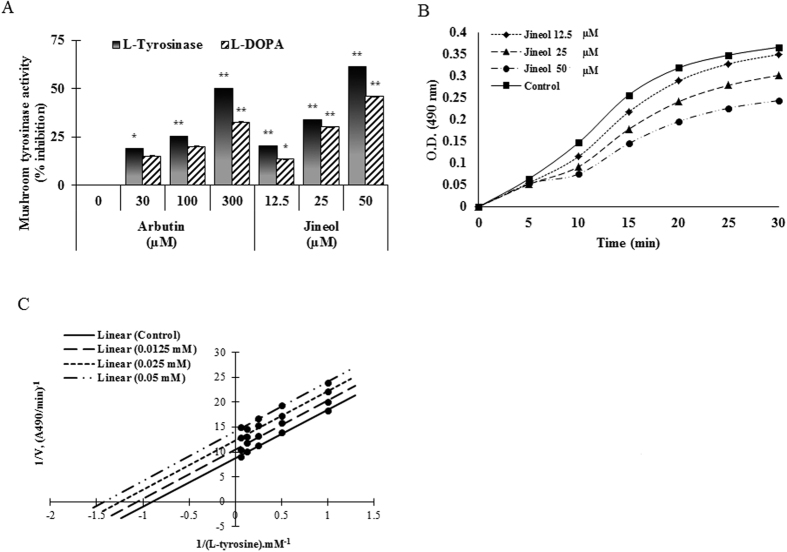
Inhibitory effects of jineol on mushroom tyrosinase activity. (**A**) Different concentrations of jineol or arbutin were incubated with the same units of mushroom tyrosinase. Following incubation, amounts of dopachrome produced were determined at 490 nm spectrophotometrically. (**B**) Effects of jineol on the monophenolase activity of tyrosinase. Enzyme activity was tested in the presence of L-tyrosine, as substrate. (**C**) Lineweaver-Burk plot of mushroom tyrosinase in the presence of jineol. Results are expressed as mean values of 1/V, as inverses of increases in absorbance at 490 nm/minute (Δ*A*_490_ per minute), and as the means of three independent tests at different L-tyrosine concentrations. Results are presented as the means ± SDs of three experiments. *P < 0.05, **P < 0.01, versus non-treated controls, Student’s t-test. Arb: Arbutin.

Mushroom tyrosinase is widely used as the target enzyme for the screening of potential inhibitors of melanogenesis, and thus, to determine whether jineol has anti-melanogenic activity, we first examined its effect on mushroom tyrosinase. The use of L-tyrosine and L-DOPA as substrates enabled us to distinguish between the ability of the compound to inhibit the o-hydroxylation of tyrosine and its further oxidation to o-diquinone. Jineol dose-dependently inhibited mushroom tyrosinase activity with an IC_50_ of 39.46 ± 0.01 and 50.35 ± 0.05 for the substrates L-tyrosine and L-DOPA, respectively, whereas arbutin (a well-known tyrosinase inhibitor) had an IC_50_ of 296.63 ± 0.01 as L-tyrosine is being a substrate (Fig 3A). Furthermore, the effects of increasing concentrations of jineol on the monophenolase and diphenolase activated forms of tyrosinase are shown in Supplementary Fig. S1.

Since no kinetic study of this inhibitory effect has been carried out prior to the current study, therefore, to evaluate the tyrosinase inhibitory effect of jineol, we tested its effect on the mono- and diphenolase activities of the enzyme by determining the kinetic parameters (Fig. 3B). When the enzymatic reaction was started by the action of tyrosinase on L-tyrosine, a marked lag period, as a characteristic of monophenolase activity^18^, was observed, simultaneously with the appearance of the first stable product, dopachrome (Fig. 3B). Interestingly, jineol behaved as an inhibitor of the monophenolase activity of tyrosinase. The lag period depended on both enzyme and substrate concentrations in the reaction medium and was shortened, or even abolished, by the presence of catalytic amounts of transition metal ions or o-diphenols^19^. However, the lag phase is known to be extended by some monophenolase inhibitors, such as glabrene and p-alkoxybenzoic acid^19^,^20^. Jineol extended the lag phase by 10 min compared to the control, especially when its concentration was 50 μM. To obtain further information about the type of inhibition exerted by jineol on mushroom tyrosinase, monophenolase activities were measured as a function of increasing concentration of L-tyrosine. The Lineweaver-Burk double-reciprocal plot obtained (Fig. 3C) showed that jineol is an uncompetitive inhibitor of the monophenolase activity of tyrosinase. On increasing jineol concentration, both maximal velocity (*V*_*max*_) of mushroom tyrosinase activity and *K*_*m*_ values were reduced (Table 1), indicating jineol is an uncompetitive inhibitor of tyrosinase. This behavior indicated that the inhibitor binds at a site distinct from the substrate and combines with the enzyme-substrate complex (ES) but not with the free enzyme (E). The equilibrium constant for inhibitor binding with enzyme-substrate complex (ES), *K*_IS_ was obtained from a plot of the vertical intercept (*1/V*m) versus the concentration of jineol which was linear (data not shown), and the obtained inhibition constant was 103 μM.

### Hypopigmentary effects of jineol on melan-a cells

**Figure 4:**
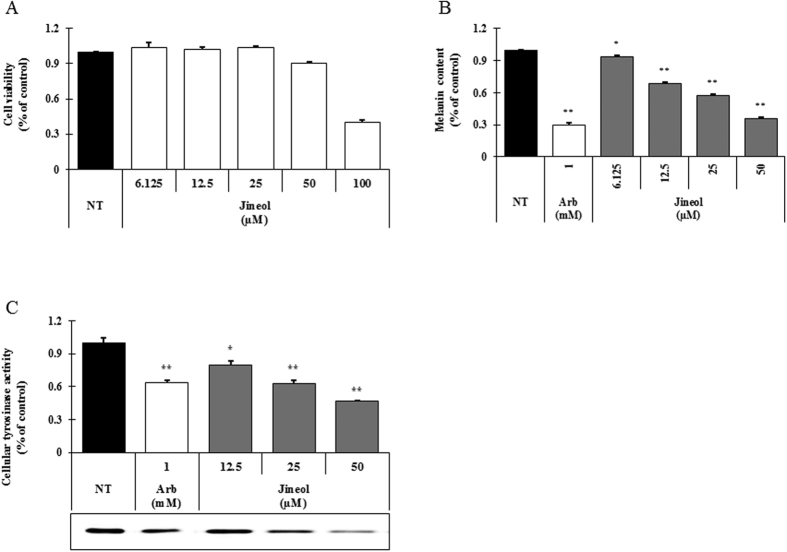
Effects of jineol on melanogenesis in melan-a cells. Cells were cultured with jineol (6.125–50 μM) for 3 days. (**A**) cytotoxicities, (**B**) melanin contents, and (**C**) tyrosinase activities were measured as described in Materials and Methods. Experiments were performed in triplicate, and results are presented as means ± SDs. *p < 0.05, **p < 0.01, student’s t-test. NT: No treatment; Arb: Arbutin.

Melanin levels in and the cell viabilities of melan-a cells were examined after exposing them to jineol at concentrations ranging from 6.125–50 μM, which exhibited no cytotoxic effect (Fig. 4A). We found that melanin contents were significantly and dose-dependently reduced after exposure to jineol (Fig. 4B, 3^rd^ to 6^th^ column). To examine the mechanics behind the inhibitory effects of jineol on melanogenesis, intracellular tyrosinase activity in melan-a cells was measured more precisely by L-DOPA zymography. As shown in Fig. 4C (3^rd^ to 5^th^ column), treatment with jineol at concentrations ranging from 12.5–50 μM increased tyrosinase inhibition. Measurements of cellular tyrosinase activity showed that jineol potently inhibited tyrosinase with an IC_50_ value of 44.66±0.01 μM. Consistent with this finding, the concentration of jineol that inhibited L-DOPA zymography band density by 50% was 46.25 μM in melan-a cells. These results suggest that jineol inhibits melanogenesis in melan-a cells.

### Effects of jineol on the expressions of melanogenesis-related proteins

**Figure 5:**
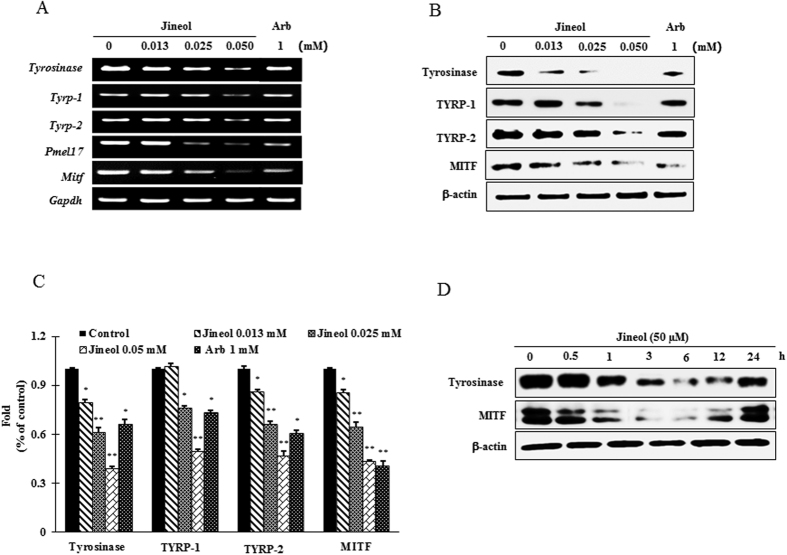
Effect of jineol on the levels of melanogenesis-related mRNA and proteins in melan-a cells. (**A**) Cells (5 × 10^5^ cells/ml) were cultured for 24 h, and the medium was then replaced with fresh medium containing the indicated concentrations of jineol or arbutin for 24 h. mRNA was extracted using TRIzol and mRNA expressions was analyzed by RT PCR. (**B**) Cells (1 × 10^5^ cells/ml) were cultured for 24 h; medium was replaced with fresh medium containing the indicated concentrations of jineol or arbutin for 3 days. Total cell lysates were extracted and assayed by western blotting using antibodies against tyrosinase, TYRP-1, TYRP-2, and MITF. Equal protein loadings were confirmed using β-actin. Arb: Arbutin. (**C**) Statistical analysis of the band intensity of tyrosinase, TYRP-1, TYRP-2, and MITF obtained by western blot analysis. *P < 0.05, **P < 0.01, versus non-treated controls, Student’s t-test. (**D**) Cells (1 × 10^5^ cells/ml) were cultured for 24 h; the medium was replaced with fresh medium containing the indicated concentrations of jineol for the indicated time interval. Total cell lysates were extracted and assayed by western blotting using antibodies against tyrosinase, and MITF. Equal protein loadings were confirmed using β-actin.

To elucidate the mechanism of melanogenesis, we examined the expressions of tyrosinase, TYRP-1, TYRP-2, and MITF in melan-a cells after jineol treatment (12.5, 25, or 50 μM) for 4 days. Jineol was observed to suppress the mRNA expressions of MITF and of its downstream genes, tyrosinase, TYRP-1 and TYRP-2 (Fig. 5A). In addition, jineol at 50 μM significantly and dose-dependently reduced tyrosinase, TYRP-1, TYRP-2, and MITF protein levels versus untreated controls (Fig. 5B). Furthermore, to observe the onset and durability of the effect of jineol on melanogenesis-related proteins, time-dependent expression of tyrosinase and MITF in melan-a cells after jineol (50 μM) treatment was performed. As expected, jineol mitigated the expression of tyrosianse and MITF from 3 to 24 h with a peak level 12 h after jineol treatment (Fig. 5D). These results suggest jineol inhibits melanogenesis by suppressing the expressions of tyrosinase-related genes and of the MITF gene.

### Effect of jineol on the p38 and ERK1/2 signaling pathways

**Figure 6:**
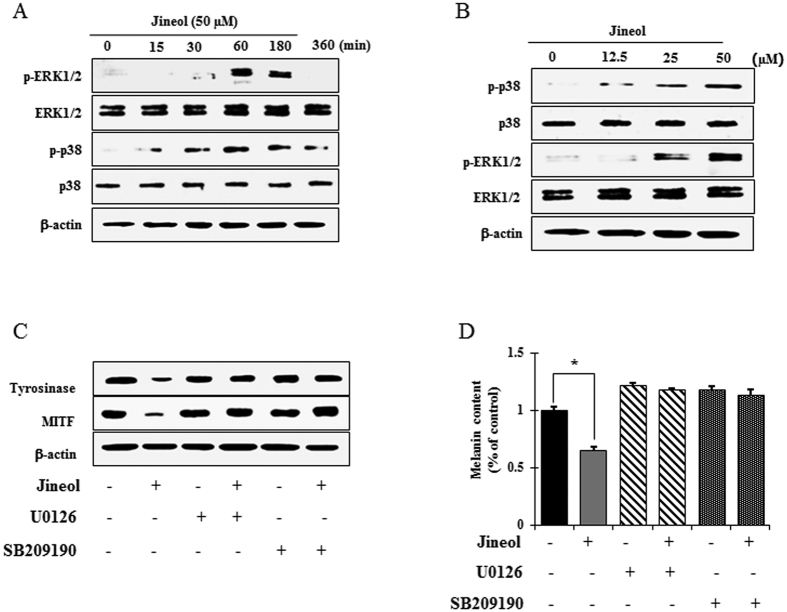
Effect of jineol on MAP kinase-dependent signaling in melan-a cells. (**A**) Cells (5 × 10^5^ cells/ml) were cultured for 24 h, and medium, then was replaced with fresh medium containing indicated concentrations of the test compounds for the indicated times. (B) Cells (5 × 10^5^ cells/ml) were cultured for 24 h, and medium, then was replaced with fresh medium containing various concentrations of the test compound for 2 h. Phosphorylations of ERK and p38 MAPK were analyzed using phospho-specific ERK and p38 MAPK antibodies. Equal protein loadings were confirmed using β-actin antibodies. (**C**) Jineol was co-treated with selective inhibitors of ERK (U0126) and p38 (SB209190) signaling molecules in melan-a cells. MITF and tyrosinase levels were analyzed by western blotting and (D) melanin contents were also determined. Determinations were made in triplicate, and results are presented as means ± SDs. *P < 0.05, **P < 0.01, versus non-treated controls, Student’s t-test.

Several signaling pathways, such as, the cAMP-dependent protein kinase (PKA), mitogen-activated protein kinase (p38, ERKs, JNK), phosphatidylinositol 3-kinase (PI3K)/AKT, and Wnt-signaling pathways modulate melanin pigment formation and melanogenic gene expressions^8^,^21^,^22^. To elucidate the mechanism underlying the melanogenic effect of jineol, melan-a cells were exposed to jineol (50 μM) for the indicated times, and protein extracts were then analyzed by western blot analysis. As shown in Fig. 6A, jineol elicited the dose-dependent phosphorylations of p38 and ERK1/2 after 60 min to 3 h of treatment (Fig. 6B). In contrast, JNK was not affected by jineol treatment in melan-a cells (data not shown). These results suggest that suppression of melanogenesis by jineol is related to p38 and ERK signaling. To determine whether increased phosphorylations of p38 and ERK were involved in the inhibition of MITF expression, following its downstream tyrosinase expression, cells were pretreated with selective inhibitors of the p38 (SB209190) or ERK (U0126) pathways before administering jineol. Treatment with the selective inhibitors of the either inhibitor abolished jineol-initiated anti-melanogenic processes via MITF inactivation and the inhibition of tyrosinase expression (Fig. 6C). To further investigate the effects of the jineol-induced phosphorylations of p38 and ERK on melanin production, melanin contents were evaluated in the presence of SB209190 and U0126 in jineol-treated cells. As was expected, melanin down-regulation by jineol was prevented by SB209190 and by U0126 (Fig. 6D). These results indicate that p38 and ERK pathway activations are involved in the jineol-induced attenuation of melanogenesis.

### Effect of jineol on proteasomal and lysosomal degradation

**Figure 7:**
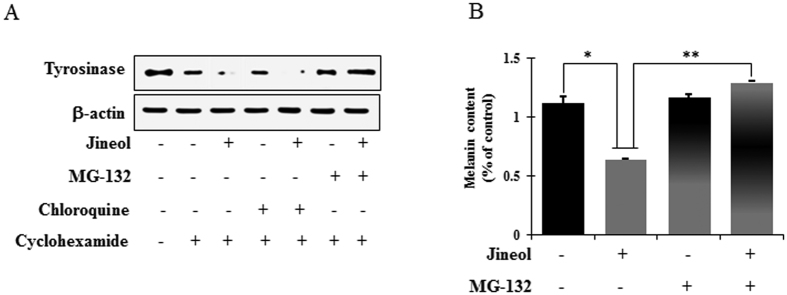
Effect of jineol on the lysosomal and proteosomal degradations of tyrosinase in melan-a cells. (**A**) Cells (3 × 10^5^ cells/ml) were pretreated with 25 μg/ml of cycloheximide (a protein synthesis inhibitor) for 1 h. Separately, cells were pretreated with 10 μM MG-132 (a proteasomal inhibitor) or 50 μM chloroquine (lysosomal degradation inhibitor) for 1 h. These cycloheximide, MG-132, or chloroquine pretreated cells were then treated with jineol for 6 h. Whole cell lysates were subjected to western blot analysis using anti-tyrosinase antibodies. Equal protein loadings were confirmed using β-actin antibodies. (**B**) Melanin contents were determined in triplicate, and results are presented as means ± SDs. *P < 0.05, versus non-treated controls, Student’s t-test.

Balance between the synthesis and degradation of tyrosinase are tightly coupled to its regulation of melanin biosynthesis, and the proteasomal and lysosomal degradations of tyrosinase have been reported to involve in the turnover of tyrosinase^9^. Thus, to investigate whether the jineol-induced downregulation of tyrosinase is associated with its post-translation degradation, we used MG-132 (a proteasome inhibitor) and/or chloroquine (a lysosomal proteolysis inhibitor). To inhibit protein synthesis, melan-a cells were pretreated with cycloheximide and MG-132 and/or chloroquine for 1 h, and this was then followed by 6 h of treatment with jineol. Western blot analysis was performed to analysis tyrosinase levels. Jineol-induced decreases in tyrosinase level were prevented by MG-132, whereas chloroquine had no effect (Fig. 7A). Intriguingly, MG-132 pretreatment prevented the suppression of melanin by jineol (Fig. 7B). Taken together, these findings demonstrated that jineol may mitigate melanin synthesis by downregulating tyrosinase by proteasomal degradation rather than by lysosomal degradation.

## Discussion

The color of human skin and hair is determined by a number of factors, and melanin biosynthesis (melanogenesis) is probably the most important. Melanogenesis is a multistage process involving melanin synthesis, melanin transport, and melanosome release^23^. Although appropriate melanogenesis provides effective protection against UV, abnormal melanin production and accumulation lead to various dermatological disorders^24^. Researchers have developed various biological agents, such as, hydroquinone, to ameliorate hyperpigmentary disorders and complexion discolorations. However, whitening products with potent tyrosinase inhibitory activity, such as, kojic acid, arbutin, and hydroquinone, have severe side effects that include vitiligo, skin peeling, and redness, and thus, their applications are limited^10^,^11^,^25^. Medicinal chemists continue to search for inhibitors of melanin production for the treatment of hyperpigmentary disorders, such as, melasma, freckles, and age spots^26^. However, because of the potential side effects of synthetic agents, natural products that inhibit the effect on melanin hyperpigmentation are viewed by some as more appropriate cosmetic agents. In the present study, jineol isolated from *Scolopendra subspinipes mutilans* was observed to inhibit mushroom tyrosinase activity significantly in an uncompetitive inhibitory manner. Furthermore, jineol also suppressed cellular melanin production by inhibiting cellular tyrosinase activity, and significantly abolished the expressions of melanogenesis-related proteins at the transcriptional and translational levels in melan-a cells.

Antioxidants protect cells from oxidative stress, and antioxidant capacity may be defined as the ability to scavenge free radicals and reactive oxygen and nitrogen species by donating hydrogen or an electron. Antioxidants are well known to play pivotal roles in the inhibition of melanogenesis in B16 cells^27^. Therefore, the antioxidative capacity of jineol on the basis of DPPH-, and ABTS-radical scavenging activity assays (Fig. 2) gives a better paradigm. This can be compared by Pearson’s correlation analysis between antioxidant and anti-melanogenic potentials (data not shown).

Tyrosinase plays a pivotal role in the melanin synthesis as it converts L-tyrosine to L-DOPA and oxidizes L-DOPA to dopachrome, and mushroom tyrosinase is widely used as a target enzyme for the screening of potential inhibitors of melanogenesis^28^. In the present study, jineol was found to significantly inhibit the both mono- and diphenolase activities of mushroom tyrosinase and the inhibitory type was classified as uncompetitive (Fig. 3). Previous study^19^, reported that tyrosinase had two sites of combinations. One for the substrate and the other for the inhibitor and jineol may just combine with the enzyme-substrate complex, but was not able to bind free enzyme molecules directly. On the basis of the results obtained, possibly the combination site of the substrate is not just for substrate, however, it can also combine with the inhibitor, but a different site may be only for the inhibitor^19^. To investigate the inhibitory effect of jineol on melanogenesis, melanin content and intracellular tyrosinase activity were performed. As shown in Fig 4B, jineol potently inhibited melanin production in melan-a cells, and in-line with this, jineol also dose-dependently suppressed intracellular tyrosinase activity (Fig. 4C).

Most tyrosinase inhibitors control melanin production by suppressing the transcriptions and translations of melanin-related proteins, such as, tyrosinase, TYRP-1, TYRP-2, ASIP (agouti signaling protein), and MGRN1 (mahogunin ring finger-1)^29^. Hence, we investigated the effects of jineol on the transcriptions and translations of melanin-related protein in melan-a cells. As was expected, jineol significantly reduced the expressions of MITF (known to be deeply involved in melanogenesis), and of enzymes downstream of MITF, such as, tyrosinase, TYRP-1, and TYRP-2 at the transcriptional and translational levels (Fig. 5). These results suggested that jineol reduces melanogenesis by inhibiting the expressions of tyrosinase, TYRP-1, and TYRP-2 by inactivating MITF in melan-a cells.

Previous studies have also shown that the MAP kinase family members, ERK and p38, play important regulatory roles during melanogenesis^30^. Studies have demonstrated that inhibitors of melanogenesis activate the phosphorylations of ERK and p38 and that these phosphorylations result in the phosphorylation of MITF at serine 73 and subsequent ubiquitin-dependent proteasomal degradation^31^. Thus, to identify the mechanisms underlying the anti-melanogenic activity of jineol, we examined MAPK signaling molecule levels in melan-a cells by western blotting. At non-toxic concentrations, jineol time-dependently activated the phosphorylations of ERK and p38, and co-treatment with jineol and an ERK1/2 inhibitor or a p38 inhibitor significantly blocked MITF suppression by jineol and eventually reduced melanin contents (Fig. 6).

The selective elimination of proteins plays and importantly regulates physiological processes in eukaryotic cells, and proteolysis in proteasomes and lysosomes are considered the major pathways of protein degradation^32^. After the post-Golgi stage, tyrosinase might be degraded in proteasomes via post-translation modification in the ER, as has been reported in linoleic acid treated melanoma cells^33^ and/or it could be degraded via the endosomal/lysosomal degradation system, as reported in inulavosin-treated melanoma cells^34^. Indeed, the degradation of tyrosinase protein is accelerated by a compound by one of the two mechanisms, considered as an anti-melanogenic compound. In the present study, the jineol-induced inhibition of tyrosinase was blocked by MG-132 in melan-a cells, which revealed that jineol-induced tyrosinase downregulation was attributable to the proteasomal degradation of endogenous tyrosinase (Fig. 7).

Previous studies revealed that the 5-HT2c receptor of 8-methylquinoline acts as a potent MCHR1 antagonist^35^. In addition, 4-substituted amino quinoline derivatives, such as, chloroquine and quinine, have been reported to inhibit melanogenesis and to involve disturbance of tyrosinase family member trafficking^36^. Furthermore, quinoline derivatives are viewed as potential whitening agents for cosmetics and as treatments for pigmentation disorders^36^.

## Conclusions

The present study describes for the first time the inhibitory effects of jineol on melanogenesis in melan-a cells. The noteworthy aspects of the present study are as follows: (a) Jineol was found to reduce melanogenesis potently, and inhibit mushroom tyrosinase activity, and to act as an antioxidant. (b) The mechanisms through which jineol mitigated melanin production involved transcription factors and the common signaling pathways of melanin synthesis. Specifically, jineol reduced melanin content in melan-a cells by downregulating MITF expression through interference with ERK1/2 and p38 phosphorylation, and decreased the protein levels of tyrosinase, TYRP-1 and TYRP-2. (c) Furthermore, our findings suggest the observed suppressive effect of jineol on melanin might be due to the proteasomal degradation of endogenous tyrosinase in melan-a cells. Based on these findings and the finding that jineol exhibited no cytotoxic activity in the present study, we suggest jineol be considered a potentially useful natural depigmentation agent.

## Materials and Methods

### Insect material

Dried *Scolopendra subspinipes mutilans* specimens were purchased at a herbal market at Geumsan, South Korea, and identified by one of the authors (M. Na). A voucher specimen (CNU-INS 1408) was deposited at the Pharmacognosy Laboratory of the College of Pharmacy, Chungnam National University (Daejeon, South Korea).

### Extraction, isolation, and characterization of jineol

Jineol was isolated from specimens using a chromatographic approach^16^. Briefly, The dried *S. subspinipes mutilans* (521 g) were extracted with ethanol (5 L × 4) at room temperature and the extract obtained was concentrated under vacuum to yield a brownish ethanol extract (110.0 g). The dried ethanol (EtOH) extract (110.0 g) was suspended in water (3 L) and fractionated successively with ethyl acetate (EtOAc, 3 L × 3) and then *n*-butanol (BuOH, 3 L × 3) to yield EtOAc-soluble (60.0 g) and *n*-BuOH-soluble (8.0 g) fractions, and residue (40.0 g). The EtOAc-soluble fraction was subjected to vacuum-liquid chromatography (VLC, 25 × 12 cm) using hexane-EtOAc 40:1, 20:1, 10:1, and 4:1; hexane-EtOAc-MeOH 2:1:0.2; CHCl_3_-MeOH 6:1; CHCl_3_-MeOH-H_2_O 3:1:0.1 and then washed with MeOH to yield 8 fractions (E1-E8). Fraction E4 (8.0 g), which was obtained by eluting with hexane-EtOAc 4:1 and was partitioned with hexane (100 mL) and MeOH (100 mL × 3) to afford jineol (60.0 mg) by checking TLC eluting with hexane-EtOAc (1:5, v/v) (*R*_*f*_ value 0.3).

Jineol: yellowish amorphous powder; ^1^H NMR (300 MHz, CD_3_OD) δ_H_ 8.46 (1 H, d, *J* = 2.4 Hz, H-2), 7.42 (1 H, d, *J* = 2.4 Hz, H-4), 7.29 (1 H, t, *J* = 8.0 Hz, H-6), 7.14 (1 H, d, *J* = 8.0 Hz, H-5), 6.87 (1 H, d, *J* = 8.0 Hz, H-7), ^13^C NMR (75 MHz, CD_3_OD) δc 154.2 (C-8), 153.0 (C-3), 142.3 (C-2), 134.8 (C-8a), 131.8 (C-4a), 129.0 (C-6), 117.9 (C-5), 117.2 (C-4), 109.0 (C-7)^17^.

### Drugs and chemicals

Arbutin, 2,2-diphenyl-1-picrylhydrazyl (DPPH), L-tyrosine, L-DOPA (L-3,4-dihydroxy phenylalanine), ascorbic acid, Tween-20, thiazolyl blue tetrazolium bromide (MTT), 2,2′-azino-bis(3-ethylbenzthiazoline-6-sulfonic acid) (ABTS), O-tetradecanoyl phorbol-13-acetate (TPA), and mushroom tyrosinase were obtained from Sigma-Aldrich (St. Louis, MO). All other reagents and chemicals were of high-grade and acquired from commercial sources. The following antibodies were purchased from Bioworld Technology (St. Louis Park, MN, USA); anti-Tyr (Cat. No. BS6754), anti-TYRP-1 (Cat. No. sc-25543), anti-TYRP-2 (Cat. No. BS3320), anti-MITF (Cat. No. BS1550) for western blotting; or Cell Signaling Technology (Beverly, MA, USA); anti-phospho-JNK (Cat. No. 4668), anti-JNK (Cat. No. 9252), anti-phospho-p38 (Cat. No. 4511), anti-p38 (Cat. No. 9212), anti-p44/42MAPK (ERK1/2) (Cat. No. 9102), and anti-phospho-p44/42MAPK (ERK1/2) (Cat. No. 4376).

### Cell culture and cell viability assay

Melan-a cells (a melanocyte cell line) were purchased from Dorothy C. Bennett (St George’s, University of London, London), and cultured in RPMI 1640 medium supplemented with 10% fetal bovine serum (FBS, Hyclone, Utah, UT, USA), streptomycin-penicillin (100 μg ml^−1^ each), and 200 nM TPA (a potent tumor promoter) at 37 °C in 5% CO_2_. Cells were passaged every 3 days up to 40 times. Confluent melanocyte monolayers were harvested using a mixture of 0.05% trypsin and 0.53 mM EDTA (Gibco BRL, Grand Island, NY, USA). A tetrazolium dye colorimetric test (MTT) was used to determine cell viabilities. Briefly, cells were first cultured in 96-well plates (1 × 10^5^ cells well^−1^) for 24 h, washed twice with phosphate buffer saline (PBS), and pre-treated with different concentrations of jineol for 24 h. MTT reagent was then added to each well and plates were re-incubated at 37 °C for 1 h. Media were then removed and plates were washed twice with PBS (pH 7.4). The formazan produced was then dissolved in 100% dimethyl sulfoxide (DMSO), and of cell or well absorbances were measured at 570 nm using a microplate reader (Victor3, PerkinElmer, Waltham, MA, USA). Percentage viabilities were calculated versus non-treated controls^2^.

### *In vitro* antioxidant assays

A DPPH (2,2-diphenyl-1-picrylhydrazyl) radical-scavenging assay was used to evaluate the free radical scavenging activity of jineol, as previously described by Nanjo *et al*.^37^, with minor modification. Briefly, a 198 μl of a 0.2 mM solution of DPPH in 50% ethanol was added to 2 μl aliquots of solutions containing different concentrations of jineol. Mixtures were allowed to stand at 25 °C for 10 min and absorbances were measured at 517 nm in a multi-label counter (Victor3, PerkinElmer, Waltham, MA, USA). Ascorbic acid was used as the standard antioxidant. Inhibitory ability (%) was calculated using the following equation:


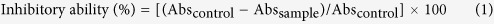


Abs_control_ = absorbance of the control; Abs_sample_ = absorbance of the test sample. All samples were analyzed in triplicate.

The method described by Noguchi *et al*.^38^ was adopted for the 2,2′-azinobis-(3-ethylbenzothiazoline)-6-sulfonic acid (ABTS) assay with slight modification. Briefly, different concentrations of jineol (2 μl) were allowed to react with 198 μl of ABTS^•+^ solution, and absorbances were determined at 734 nm. Ascorbic acid used as the standard. Inhibitory ability (%) was calculated using Equation 1.

A ferric reducing antioxidant power (FRAP) assay was used to measure reducing power, as described previously^39^, with a slight modification. Ascorbic acid was used as the standard antioxidant, and ascorbic acid equivalent FRAP values (μM) were determined using an ascorbic acid standard curve.

Cupric-reducing antioxidant capacity (CUPRAC) of jineol was assayed as described by Apak *et al*.^40^, with slight modification. Ascorbic acid equivalent CUPRAC values (μM) were also determined using ascorbic acid standard curve.

### Measurement of mushroom tyrosinase activity

Tyrosinase activity was determined spectrophotometrically as previously described, with minor modification^2^. Briefly, mixtures of 0.1 M phosphate buffer (pH 6.5) (100 μl), 1 mM L-tyrosine (50 μl), mushroom tyrosinase (EC 1.14.18.1; 200 units ml^−1^ in phosphate buffer, pH 6.5) with or without jineol at different concentrations were prepared in a 96-well microplate (SPL, Pocheon, Korea). After recording initial absorbances at 490 nm using a microplate reader (VICTOR3, Perkin Elmer), the plate was incubated at 37 °C for 30 min, and absorbances were re-recorded. Tyrosinase inhibitory activity was calculated using the following equation:


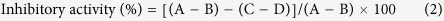


where A is the final absorbance of the control (without jineol), B is the initial absorbance of the control, C is the final absorbance in the presence jineol, and D is the initial absorbance in the presence of jineol.

### Measurement of cell melanin contents

Melan-a cells (1 × 10^5^ cells ml^−1^) were seeded into a 24-well plate (BD Falcon, Bedford, MA, USA) and allowed to attach overnight. Medium was then replaced with fresh media containing different concentrations of jineol and cultured for a further 72 h. Cells were then washed twice with PBS, lysed with 1 N NaOH, transferred to a 96-well plate, and melanin contents were measured at 405 nm using a microplate reader (VICTOR3, Perkin Elmer). Arbutin was used as the positive control^41^. Inhibition of melanogenesis (%) was calculated using the following equation:


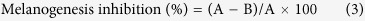


where A is the absorbance of cells treated with jineol or arbutin and B is the absorbance of non-treated control cells.

### Measurement of tyrosinase activity by zymography

Tyrosinase zymography was performed as described previously^42^. Cells (1 × 10^5^ cells ml^−1^) were cultured with or without jineol for 72 h, washed with PBS twice, and harvested using RIPA cell lysis buffer supplemented with protease and phosphatase inhibitors. A BCA protein assay kit (Pierce Biotechnology, Rockford, IL) was used to measure total protein concentration. Total proteins (50 μg) were separated by 10% SDS-PAGE (sodium dodecyl sulfate-polyacrylamide gel electrophoresis), and gels then incubated in 0.1 M sodium phosphate buffer for 30 min with a mild shaking, and stained with 20 mM L-DOPA in 0.1 M sodium phosphate buffer at 37 °C for 1 h. Intracellular tyrosinase was detected by treating gels with L-DOPA solution.

### Reverse transcription-polymerase chain reaction (RT-PCR)

Total RNA was extracted from melan-a cells using TRIzol (Ambion, Austin, TX, USA), according to the manufacturer’s instructions^43^. To prepare a cDNA pool from RNAs, total RNA (2 μg) was transcribed using an RT-&GO Mastermix (MP Biomedicals, Seoul, Korea), and the product was used as the PCR template. Reverse transcription PCR (RT-PCR) was performed using a PCR Thermal Cycler Dice TP600 (TAKARA Bio Inc., Otsu, Japan) using the following primer sequences: *tyrosinase* (forward, 5′-CCCAGAAGC CAATGCACCTA-3′, reverse, 5′-ATAACAGCTCCCACCAGTGC-3′); mouse *TYRP-1* (forward, 5′-GCTGCAGGAGCCTTCTTTCT-3′, reverse, 5′-AGACGCTGCACTGCTGGTC-3′); mouse *TYRP-2* (forward, 5′-GGATGACCGTGAGCAATGGC-3′, reverse, 5′-CGGTTG TGACCAATGGGTGC-3′); mouse *MITF* (forward, 5′CAGGCTAGAGCGCATGGACT-3′, reverse, 5′-CTCCGTTTCTTCTGCGCTCA-3′); and mouse glyceraldehyde-3-phosphate dehydrogenase (*GAPDH*) as an internal control (forward, 5′-GC GAGACCCCACTAACATCA-3′, reverse, 5′-GAGTTGGGATAGGGCCTCTCTT-3′). PRC products were separated in 2% agarose gel in Tris-Acetate-EDTA (TAE) buffer at 100 V for 30 min and visualized by ethidium bromide staining.

### Preparation of cell lysates and western blotting

Melan-a cell lysates were prepared using a standard protocol, mixed with sample buffer (250 mM Tris-HCl (pH 6.8), 0.5 M DTT, 10% SDS, 0.5% bromophenol blue, 50% glycerol, 5% 2-mercaptoethanol) and denatured at 100 °C for 5 min. Sample proteins (20 μg) were separated by 10% SDS-PAGE and electrotransferred to nitrocellulose membranes (Whatman, Dassel, Germany). Membranes were incubated overnight with 5% skim milk with primary antibodies, that is, anti-tyrosinase (C-19), anti-TYRP1 (G-17), anti-TYRP2 (D-18), anti-MITF, and β-actin (Bioworld Technology, St. Louis Park, MN, USA). Anti-goat IgG-horse radish peroxidase (HRP) (Santa Cruz) and anti-mouse IgG-HRP (Santa Cruz) were used as secondary antibodies. The antigen-antibody reaction was detected using an ECL solution system (Perkin Elmer).

### Statistical analysis

Results were analyzed using one-way ANOVA, and are presented as means ± SDs. The analysis was performed using SPSS for Windows Ver. 10.07 (SPSS, Chicago, IL, USA), and statistical significance was accepted for p values of p < 0.01 or <0.05 as indicated.

## Additional Information

**How to cite this article:** Alam, M. B. *et al*. Inhibition of melanogenesis by jineol from *Scolopendra subspinipes mutilans* via MAP-Kinase mediated MITF downregulation and the proteasomal degradation of tyrosinase. *Sci. Rep.* **7**, 45858; doi: 10.1038/srep45858 (2017).

**Publisher's note:** Springer Nature remains neutral with regard to jurisdictional claims in published maps and institutional affiliations.

## Supplementary Material

Supplementary Dataset 1

## Figures and Tables

**Table 1:** Kinetic parameters of mushroom tyrosinase in the presence of jineol.

Compound	Concentration (M)	Km (M)	V_max_ (Δ*A*_490_ per min)
None	—	8.5 × 10^−4^	8.7 × 10^−2^
Jineol	1.2 × 10^−5^	10.3 × 10^−4^	10.4 × 10^−2^
	2.5 × 10^−5^	12.2 × 10^−4^	12.3 × 10^−2^
	5.0 × 10^−5^	14.0 × 10^−4^	14.0 × 10^−2^
