# Supplementary material for: Inhibition of melanogenesis by jineol from Scolopendra subspinipes mutilans via MAP-Kinase mediated MITF downregulation and the proteasomal degradation of tyrosinase
Source: Sci Rep. 2017 Apr 10;7:45858. doi: 10.1038/srep45858 (PMC5385534; doi:10.1038/srep45858)
Supplement: Supplementary Dataset 1 [file srep45858-s1.doc]

**Supplementary Data Set 1**

**Inhibition of melanogenesis by jineol from *Scolopendra subspinipes mutilans* via MAP-Kinase mediated MITF downregulation and the proteasomal degradation of tyrosinase**

**Md Badrul Alam^1,#^, Vivek K. Bajpai^2,#^, JungIn Lee^3^, Peijun Zhao^1^, Jung-Hee Byeon^1^, Jeong-Sic Ra^1^, Rajib Majumder^4,5^, Jong Sung Lee^6^, Jung-In Yoon^6^, Irfan A. Rather^2^, Yong-Ha Park^2^, Kangmin Kim^7,*^, MinKyun Na^3,*^, Sang-Han Lee^1,*^**

^1^Department of Food Science and Biotechnology, Graduate School, Kyungpook National University, Daegu 41566, Korea

^2^Department of Applied Microbiology and Biotechnology, Microbiome Laboratory, Yeungnam University, Gyeongsan, Gyeongbuk 38541, Korea

^3^College of Pharmacy, Chungnam National University, Daejeon 34134, Korea

^4^Department of Biological Sciences, Macquarie University, Sydney, NSW 2109, Australia

^5^Bio-security and Food Safety, NSW Department of Primary Industries, Elizabeth Macarthur Agricultural Institute (EMAI), Menangle, NSW 2567, Australia

^6^Kcellbio, Seoulsoop Kolon Digital Tower, Seongsuil-ro-4-gil, Seongdong-gu 04713, Seoul, Korea

^7^Division of Biotechnology, College of Environmental and Bioresource Sciences, Chonbuk National University, 79 Gobong-ro, Iksan-si 570-752, Jeonbuk, Republic of Korea

**^#^These authors contributed equally to this work**

***Correspondence to:**

Dr. Kangmin Kim; E-mail: [activase@jbnu.ac.kr](mailto:activase@jbnu.ac.kr) Tel: +82-63-850-0834

Dr. MinKyun Na; E-mail: [mkna@cnu.ac.kr](mailto:mkna@cnu.ac.kr); Tel: +82-42-821-5925

Dr. Sang-Han Lee; Email: [sang@knu.ac.kr](mailto:sang@knu.ac.kr); Tel: +82-53-950-7754


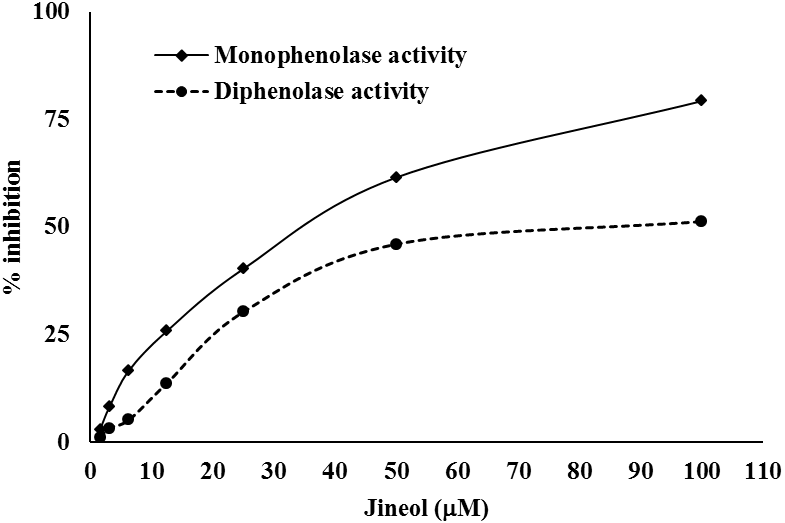


**Figure S1:** Effect of jineol on the monophenolase and diphenolase activities of mushroom tyrosinase. Inhibition of L-tyrosine and L-DOPA oxidation to dopachrome by jineol was measured at 490 nm. Distilled water was used as a control. Data presented as percent inhibition.
